# Hypertonia-linked protein Trak1 functions with mitofusins to promote mitochondrial tethering and fusion

**DOI:** 10.1007/s13238-017-0469-4

**Published:** 2017-09-18

**Authors:** Crystal A. Lee, Lih-Shen Chin, Lian Li

**Affiliations:** 1 Department of Pharmacology, Emory University School of Medicine, Atlanta, GA 30322 USA; 2 Present Address: Cell Biology Section, Neurogenetics Branch, National Institute of Neurological Disorders and Stroke, National Institutes of Health, Bethesda, MD 20892 USA

**Keywords:** mitochondria, mitochondrial fusion, mitochondrial tethering, mitofusin, hypertonia

## Abstract

Hypertonia is a neurological dysfunction associated with a number of central nervous system disorders, including cerebral palsy, Parkinson’s disease, dystonia, and epilepsy. Genetic studies have identified a homozygous truncation mutation in Trak1 that causes hypertonia in mice. Moreover, elevated Trak1 protein expression is associated with several types of cancers and variants in Trak1 are linked to childhood absence epilepsy in humans. Despite the importance of Trak1 in health and disease, the mechanisms of Trak1 action remain unclear and the pathogenic effects of Trak1 mutation are unknown. Here we report that Trak1 has a crucial function in regulation of mitochondrial fusion. Depletion of Trak1 inhibits mitochondrial fusion, resulting in mitochondrial fragmentation, whereas overexpression of Trak1 elongates and enlarges mitochondria. Our analyses revealed that Trak1 interacts and colocalizes with mitofusins on the outer mitochondrial membrane and functions with mitofusins to promote mitochondrial tethering and fusion. Furthermore, Trak1 is required for stress-induced mitochondrial hyperfusion and pro-survival response. We found that hypertonia-associated mutation impairs Trak1 mitochondrial localization and its ability to facilitate mitochondrial tethering and fusion. Our findings uncover a novel function of Trak1 as a regulator of mitochondrial fusion and provide evidence linking dysregulated mitochondrial dynamics to hypertonia pathogenesis.

## INTRODUCTION

Mitochondria are dynamic, multi-functional organelles that are crucial for life and death of eukaryotic cells (Detmer and Chan, 2007; Parsons and Green, 2010; Nunnari and Suomalainen, 2012). Mitochondria actively undergo fusion and fission, which determine mitochondrial morphology (Twig et al., 2008; Wang et al., 2012). Proper control of mitochondrial fusion and fission is vital to mitochondrial physiology and overall cellular health (Chan, 2012). Defects in mitochondrial dynamics have been linked to a variety of human diseases, including neurodegenerative disorders (Chen and Chan, 2009; Zuchner et al., 2004; Winklhofer and Haass, 2010) and cancer (Zhao et al., 2013; Rehman et al., 2012). Mitochondrial fusion and fission are controlled by the opposing actions of different GTPases: mitofusins (Mfn1 and Mfn2) and OPA1 promote outer and inner mitochondrial membrane fusion, respectively (Chen et al., 2003; Santel and Fuller, 2001; Legros et al., 2002; Cipolat et al., 2004), while dynamin-related protein 1 (Drp1) mediates mitochondrial fission (Smirnova et al., 2001). In spite of recent progress in the study of mitochondrial dynamics, our current knowledge of the molecular mechanisms that regulate mitochondrial fusion and fission processes is incomplete.

Hypertonia, a neurological symptom which is characterized by stiff gait, abnormal posture, jerky movements, and tremor, is observed in many central nervous system disorders, including cerebral palsy, Parkinson’s disease, dystonia, stroke, and epilepsy (Sanger et al., 2003; Bar-On et al., 2015). A frameshift mutation in the *Trak1* gene that generates a C-terminal truncated form of Trak1 has been identified as the genetic defect for causing recessively transmitted hypertonia in mice (Gilbert et al., 2006). Furthermore, variants in Trak1 has been linked to childhood absence epilepsy in humans by a genome-wide high-density SNP-based linkage analysis (Chioza et al., 2009). Additionally, altered Trak1 protein expression is associated with gastric and colorectal cancers (Zhang et al., 2009; An et al., 2011) and recently, whole exome sequencing has identified pathogenic variants in Trak1 that cause human fatal encephalopathy (Barel et al., 2017). The connection of Trak1 to multiple disease states highlights the importance of understanding the functional roles of Trak1 and the pathogenic effects of its dysfunction.

Trak1 is a ubiquitously expressed protein that has been implicated in regulation of mitochondrial transport (van Spronsen et al., 2013; Stowers et al., 2002; Brickley and Stephenson, 2011) and endosome-to-lysosome trafficking (Webber et al., 2008). Studies in *Drosophila* and mammalian cells have shown that Trak1 and its *Drosophila* homologue Milton can act as adaptor proteins through interaction with the mitochondria-anchored Rho GTPase, Miro, and microtubule-based motor proteins, kinesin and dynein/dynactin, to facilitate axonal transport of mitochondria in neurons (van Spronsen et al., 2013; Stowers et al., 2002; Brickley and Stephenson, 2011; Glater et al., 2006). The functional role of Trak1 in non-neuronal cells is less understood. Furthermore, it is unclear whether Trak1 also functions in other mitochondrial processes besides regulating mitochondrial motility.

In this study, we identified a novel function for Trak1 in regulation of mitochondrial fusion and showed that Trak1 is required for stress-induced mitochondrial hyperfusion and pro-survival response. Our analyses revealed that Trak1 interacts and colocalizes with mitofusins and acts with mitofusins to promote mitochondrial tethering and fusion. We found that the mitochondrial localization of Trak1 and its ability to facilitate mitochondrial fusion is impaired by hypertonia-linked Trak1 mutation. Our findings provide new insights into the fundamental mechanisms governing mitochondrial dynamics and have important implications for understanding and treating hypertonia.

## RESULTS

### Trak1 is required for normal morphogenesis of mitochondria

**Figure 1:**
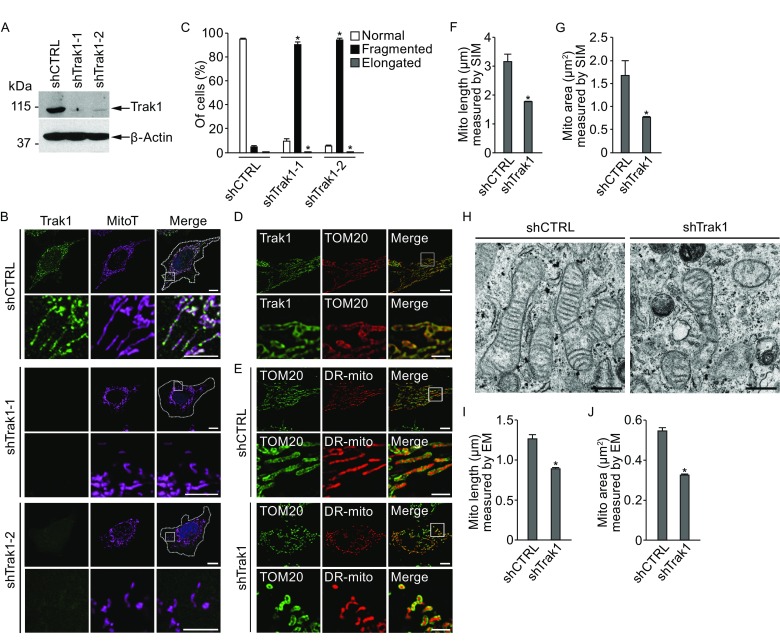
**Depletion of endogenous Trak1 alters mitochondrial morphology**. (A) Western blot analysis of cell lysates with anti-Trak1 antibody shows depletion of endogenous Trak1 protein in HeLa cells stably transfected with Trak1-targeting shRNAs (shTrak1-1 and shTrak1-2) compared with HeLa cells transfected with non-targeting control shRNAs (shCTRL). Anti-β-actin immunoblotting was used as a loading control. (B) Immunofluorescence confocal microscopic analysis with anti-Trak1 antibody (green) and MitoTracker Deep Red (MitoT; purple) shows altered mitochondrial morphology in shTrak1-transfected HeLa cells compared with the shCTRL-transfected control. The boundary of cells is indicated by the dotted line and the nuclei are visualized by DAPI stain (blue) in merged images. Enlarged view of the boxed region is shown below the original image. Scale bars: 10 μm in the original images and 5 μm in the enlarged images. (C) The percentage of cells with indicated mitochondrial morphology was quantified and shown as mean ± SEM of three independent experiments. *, *P* < 0.05 versus the shCTRL-transfected control, one-way analysis of variance with a Tukey’s *post hoc* test. (D–G) Dual-color 3D-SIM super-resolution imaging analysis of HeLa cells (D) immunostained with anti-Trak1 antibody (green) and anti-TOM20 antibody (red); and shCTRL- or shTrak1-transfected HeLa cells (E) labeled with DsRed2-mito (DR-mito; red) and anti-TOM20 antibody (green). Enlarged view of the boxed region is shown below the original image. Scale bars: 5 μm in the original images and 2 μm in the enlarged images. Mitochondrial length (F) and mitochondrial area (G) were quantified and shown as mean ± SEM of three independent experiments. In total, 382 (shCTRL) and 516 (shTrak1) mitochondria were analyzed. *, *P* < 0.05 versus the shCTRL control, unpaired two-tailed Student’s *t* test. (H–J) Electron microscopic analysis of mitochondrial ultrastructure in shCTRL- or shTrak1-transfected HeLa cells (H). Scale bars: 0.5 μm. Mitochondrial length (I) and mitochondrial area (J) were quantified and shown as mean ± SEM (*n* = 5). In total, 84 (shCTRL) and 115 (shTrak1) mitochondria were analyzed. *, *P* < 0.05 versus the shCTRL control, unpaired two-tailed Student’s *t* test

To investigate the role of Trak1 in mitochondrial regulation, we generated stably transfected HeLa cells expressing Trak1-targeting shRNAs (shTrak1) to deplete endogenous Trak1 protein. As shown in Fig. 1A, shTrak1-1 and shTrak1-2, two distinct shRNAs which target different regions of Trak1 mRNA, both effectively inhibited endogenous Trak1 protein expression. Immunofluorescence confocal microscopic analyses showed that a substantial population of endogenous Trak1 was localized to MitoTracker-labeled mitochondria in control cells (Fig. 1B). Depletion of endogenous Trak1 resulted in a loss of mitochondria at the cell periphery and accumulation of mitochondria in the perinuclear region (Fig. 1B), consistent with the previously reported function of Trak1 in mitochondrial transport (van Spronsen et al., 2013; Brickley and Stephenson, 2011; Glater et al., 2006; Brickley et al., 2005). Importantly, we found that Trak1 depletion also caused fragmentation of mitochondria into small tubules and spheres (Fig. 1B and 1C), indicating that endogenous Trak1 is required for normal morphogenesis of mitochondria.

To further characterize Trak1 depletion-induced mitochondrial morphological phenotype, we performed super-resolution imaging analyses using three-dimensional structured illumination microscopy (Huang et al., 2009; Fallaize et al., 2015). Mitochondria were visualized using the mitochondrial matrix marker DsRed2-Mito and the antibody against the outer mitochondrial membrane (OMM) protein TOM20. We found that endogenous Trak1 was localized to the OMM (Fig. 1D) and that DsRed2-Mito-labeled mitochondrial matrix was surrounded by TOM20-positive OMM (Fig. 1E). Our 3D-SIM analyses revealed that depletion of endogenous Trak1 resulted in a significant decrease in the average length and size of individual mitochondria compared with those in the control cells (Fig. 1D–G).

Next, we performed electron microscopy (EM) analyses to assess ultrastructural changes in mitochondria caused by Trak1 depletion. As shown in Fig. 1H, mitochondria from the control cells were mostly tubular in appearance with well-organized cristae structures. In contrast, mitochondria in Trak1-depleted cells were shorter and smaller, often with a spherical or oval shape and disorganized or disrupted cristae structures (Fig. 1H). In accord with the 3D-SIM results, our EM analyses indicated Trak1 depletion caused a significant reduction in the average length and size of individual mitochondria (Fig. 1H–J). The apparent mitochondrial length and area measured by EM (Fig. 1I and 1J) were notably smaller than those measured by 3D-SIM (Fig. 1F and 1G), which is likely due to differences in sample preparation/sectioning procedures and resolving powers of these two types of microscopy. Together, our results from confocal, 3D-SIM, and EM analyses of Trak1 depletion phenotype reveal a function of Trak1 in the control of mitochondrial morphology.

### Trak1 controls mitochondrial morphology by regulating mitochondrial fusion

**Figure 2:**
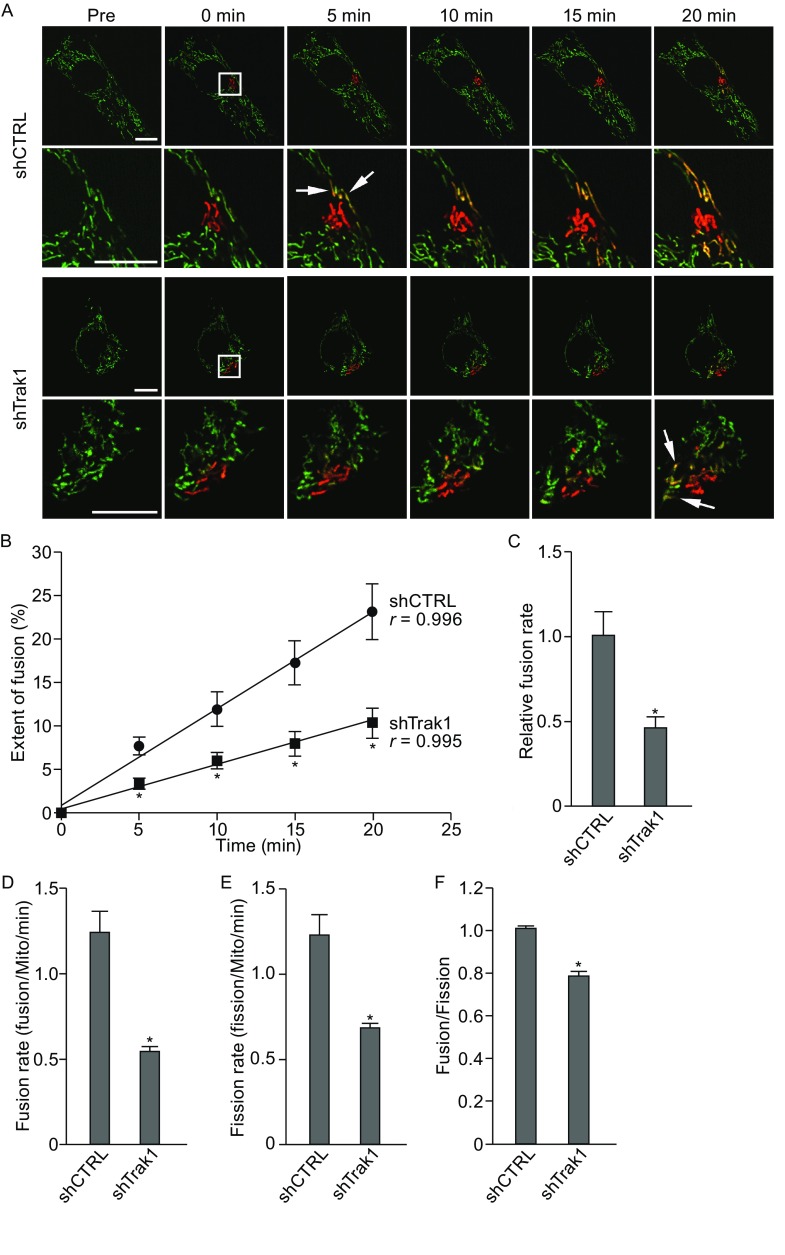
**Trak1 depletion impairs mitochondrial fusion and causes mitochondrial fusion-fission imbalance**. (A) Live-cell imaging analysis of mitochondrial fusion in stable shCTRL- or shTrak1-transfected HeLa cells labeled with mitoDendra2. Before photoactivation (Pre), mitoDendra2-labeled mitochondria emit green fluorescence. A subset of mitochondria was photoconverted from green to red by photoactivation at time 0 (boxed area). Time-lapse cell images were captured every 30 s for at least 20 min. Enlarged view of the area around the region of photoactivation is shown below the original image. Arrows indicate examples of yellow mitochondria formed by the fusion of red and green mitochondria. Scale bars: 10 μm. (B) The extent of mitochondrial fusion was assessed by quantification of colocalization to determine the percentage of the area of green mitochondria overlapping with red mitochondria at the indicated time points. (C) The relative fusion rate was determined as the slope from linear regression analysis of the data in (B) and is expressed relative to that of the shCTRL-transfected control. (D–F) Quantification of mitochondrial fusion rate (D), mitochondrial fission rate (E), and the ratio of fusion rate over fission rate (F). Data represent mean ± SEM of three independent experiments. *, *P* < 0.05 versus the shCTRL control, unpaired two-tailed Student’s *t* test

Because mitochondrial morphology is determined by the balance between fusion and fission, our finding of the fragmented mitochondrial morphology caused by Trak1 depletion (Fig. 1) raised the possibility that Trak1 may have a role in regulation of mitochondrial fusion and fission dynamics. To examine this possibility, we performed live-cell, time-lapse imaging analyses to assess the effects of Trak1 depletion on mitochondrial fusion and fission activities using mitoDendra2 as a probe. MitoDendra2 is a mitochondrial matrix-targeted, photo-switchable fluorescent protein which can be irreversibly converted from green to red fluorescent state by photoactivation (Magrane et al., 2012). The fusion between red (photoactivated) and green (non-activated) mitochondria can be detected in merged images as yellow fluorescence generated after mixing of green and red fluorescence in the matrix of fused mitochondria (Fig. 2A). Our time-lapse cell imaging analysis revealed considerably less mitochondrial fusion in Trak1-depleted cells compared to the controls (Fig. 2A). Quantitative analysis of the extent of mitochondrial fusion by measuring the colocalization between mitochondrial green and red fluorescence showed a linear increase (correlation coefficient *r* ≥ 0.99) in the extent of fusion over time in both control and Trak1-depleted cells (Fig. 2B), but the relative fusion rate estimated from the slope of the linear regression analysis was reduced by 54.4% ± 2.9% by Trak1 depletion (Fig. 2B and 2C). When mitochondrial fusion rate was quantified as the number of individual fusion events per mitochondria per min, there was 55.3% ± 3.5% decrease in the fusion rate in Trak1-depleted cells compared to the control cells (Fig. 2D). Together, these results indicate that the fragmented mitochondrial morphology observed in Trak1-depleted cells (Fig. 1) is attributable at least in part to the reduced mitochondrial fusion rate caused by Trak1 depletion.

In addition to the reduced mitochondrial fusion rate, increased mitochondrial fission rate may also contribute to the mitochondrial fragmentation phenotype induced by Trak1 depletion. To address this issue, we measured mitochondrial fission rate by quantifying the number of individual fission events per mitochondria per min in Trak1-depleted cells and their controls. We found that Trak1 depletion resulted in a significant decrease rather than an increase in the mitochondrial fission rate (Fig. 2E), thus excluding the possibility of enhanced fission activity as a cause of the observed fragmented mitochondrial morphology. The decreased mitochondrial fission rate in Trak1-depleted cells is likely a secondary effect resulting from the decreased mitochondrial fusion rate induced by Trak1 depletion, as accumulating evidence indicates that altered fusion rate can lead to a compensatory change in the fission rate due to the close interplay between fusion and fission (Twig et al., 2008; Wang et al., 2012; Cagalinec et al., 2013). For example, reduced mitochondrial fusion rate resulted from loss of Mfn1 and/or Mfn2 was found to associate with a decrease in the mitochondrial fission rate (Wang et al., 2012).

Quantitative analysis of the ratio of fusion rate over fission rate showed that, in contrast to the control cells which have a balanced mitochondrial fusion and fission rates (Fig. 2F), the mitochondrial fusion-fission balance was impaired in Trak1-depleted cells, as Trak1 depletion caused a greater reduction (55.3% ± 3.5%) in the fusion rate than the reduction (42.8% ± 4.9%) in the fission rate (Fig. 2D–F). The imbalanced mitochondrial fusion and fission rates resulted in mitochondrial fragmentation, leading to altered mitochondrial morphology seen in Trak1-depleted cells. Together, these data support a function of Trak1 in the control of mitochondrial morphology by regulating mitochondrial fusion.

### Hypertonia-linked mutation impairs Trak1 mitochondrial localization and function

**Figure 3:**
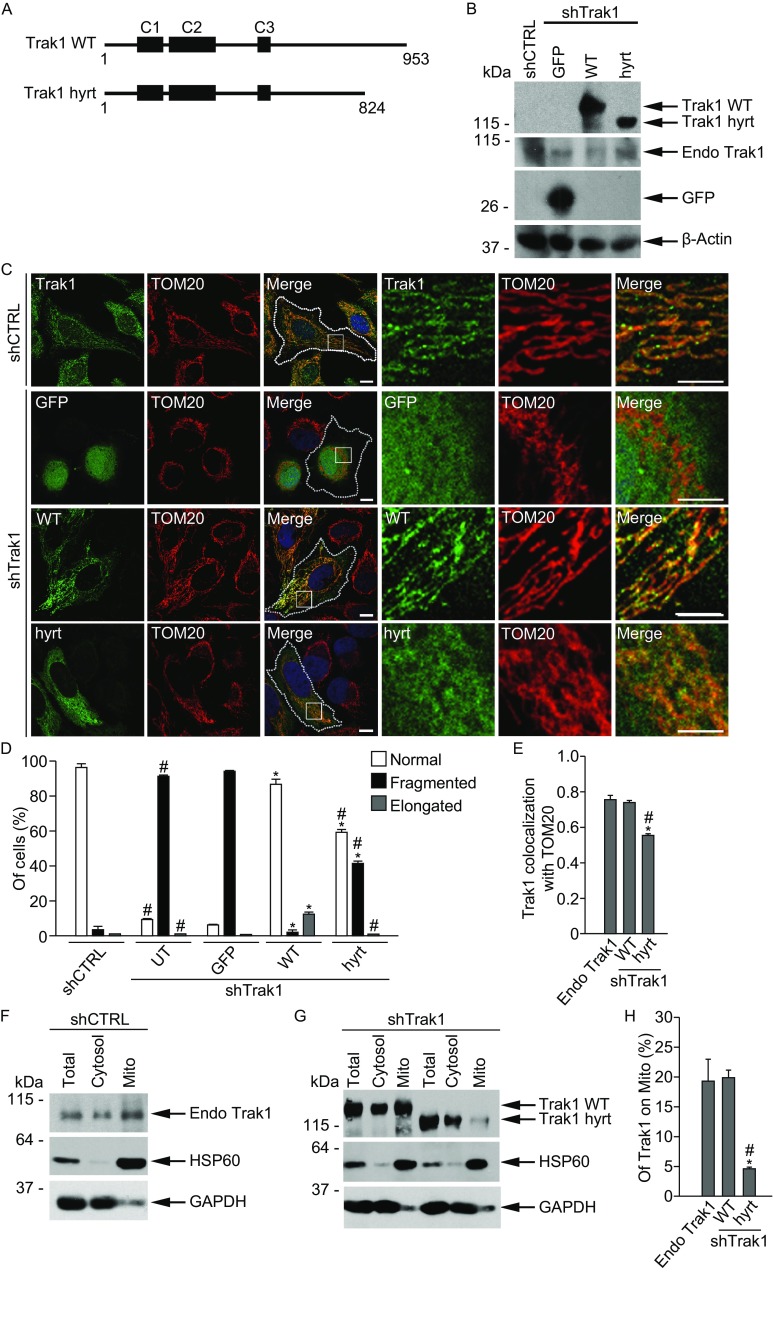
**Impairment of Trak1 mitochondrial localization and function by hypertonia-linked mutation**. (A) Schematic representation of Trak1 WT and hypertonia-associated mutant Trak1 hyrt. The locations of three coiled-coil domains, C1, C2, and C3 are indicated. (B) Trak1-depleted HeLa cells (shTrak1 cells) were “rescued” by transfection with shTrak1-resistant GFP-tagged Trak1 WT and Trak1 hyrt, or GFP control, and cell lysates were analyzed along with shCTRL control by immunoblotting with antibodies against GFP, endogenous (endo) Trak1, and β-actin. (C) Confocal microscopic analysis of shCTRL cells immunostained with anti-Trak1 antibody (green) and anti-TOM20 antibody (red); and shTrak1 cells “rescued” with the indicated GFP-tagged Trak1 protein or GFP control (green) and immunostained with anti-TOM20 antibody (red). The boundary of cells is indicated by the dotted line and nuclei visualized by DAPI stain (blue) in merged images. Enlarged view of the boxed region is shown next to the original image. Scale bars: 10 μm in the original images and 5 μm in the enlarged images. (D) The percentage of cells with indicated mitochondrial morphology was quantified and shown as mean ± SEM of three independent experiments. *, *P* < 0.05 versus the untransfected (UT); #, *P* < 0.05 versus Trak1 WT, one-way analysis of variance with a Tukey’s *post hoc* test. (E) The fraction of Trak1 colocalized with TOM20 was determined by Mander’s colocalization coefficient. Data represents mean ± SEM of three independent experiments. *, *P* < 0.05 versus endogenous Trak1; #, *P* < 0.05 versus Trak1 WT, one-way analysis of variance with a Tukey’s *post hoc* test. (F–H) Postnuclear supernatant (Total) from either untransfected shCTRL cells (F) or shTrak1 cells transfected with the indicated GFP-tagged Trak1 rescue constructs (G) were separated into cytosol and mitochondria (Mito) fractions. Aliquots representing 1% of Total or cytosol fraction and 6% of Mito fraction were analyzed by immunoblotting with antibodies against Trak1 (F), GFP (G), HSP60, and GAPDH. The percentage of Trak1 in the mitochondrial fraction (H) was quantified and shown as mean ± SEM of three independent experiments. *, *P* < 0.05 versus endogenous Trak1; #, *P* < 0.05 versus Trak1 WT, one-way analysis of variance with a Tukey’s *post hoc* test

The functional consequence of hypertonia-associated Trak1 mutation, which generates a Trak1 mutant protein truncated at amino acid 824 (Fig. 3A), remains unknown. To determine the effect of hypertonia-associated mutation on Trak1 function, we took advantage of the Trak1 depletion phenotype (Fig. 1) and performed rescue experiments by expressing shTrak1-resistant GFP-tagged full-length wild-type Trak1 protein (Trak1 WT), hypertonia-associated Trak1 mutant (Trak1 hyrt), or GFP control in Trak1-depleted cells and assessing their abilities to rescue the mitochondrial morphological defects (Fig. 3B–D). Our analyses revealed that Trak1 WT expression was able to restore the tubular mitochondrial morphology in Trak1 WT-transfected shTrak1 cells, whereas mitochondria remain fragmented in neighboring untransfected shTrak1 cells or in GFP-transfected shTrak1 cells (Fig. 3C and 3D). The ability of Trak1 WT to rescue the mitochondrial fragmentation phenotype of Trak1 depletion confirmed that the altered mitochondrial morphology in shTrak1 cells was caused specifically by the loss of Trak1 but not off-target effect of shTrak1. We found that Trak1 hyrt mutant was significantly less effective than Trak1 WT in rescuing the mitochondrial fragmentation phenotype of Trak1 depletion (Fig. 3C and 3D), indicating hypertonia-associated Trak1 mutation causes a partial loss of Trak1 function in regulation of mitochondrial morphology.

We observed that the immunostaining pattern of Trak1 hyrt mutant was consistently less mitochondrial and more diffuse compared to that of Trak1 WT or endogenous Trak1 (Fig. 3C). Quantitative analysis showed that Trak1 hyrt mutant had significantly reduced colocalization with the mitochondrial marker TOM20 than that of Trak1 WT or endogenous Trak1 (Fig. 3E), suggesting that the localization of Trak1 to mitochondria is partially impaired by hypertonia-linked Trak1 mutation. To further examine this possibility, we performed subcellular fractionation analyses to assess the relative distributions of endogenous and exogenous Trak1 proteins in cytosolic and mitochondrial fractions (Fig. 3F and 3G). We found that the percentage of Trak1 hyrt mutant associated with the mitochondrial fraction was significantly decreased compared to that of Trak1 WT or endogenous Trak1 (Fig. 3F–H), providing additional evidence for hypertonia mutation-induced impairment in Trak1 mitochondrial localization.

### Trak1 overexpression elongates and enlarges mitochondria

**Figure 4:**
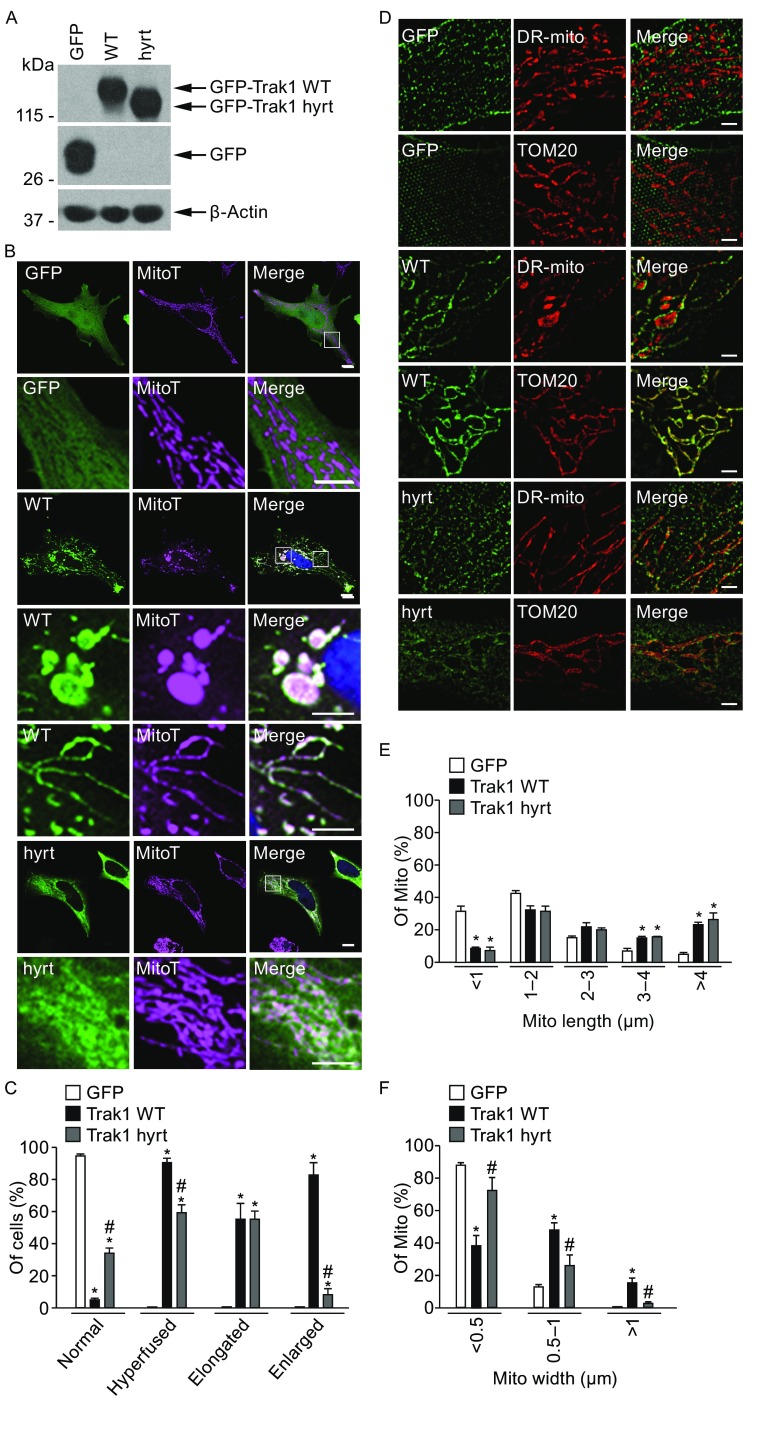
**Trak1 overexpression induces mitochondrial hyperfusion and this effect is reduced by hypertonia-linked Trak1 mutation**. (A) Western blot analysis of cell lysates with anti-GFP antibody shows expression of GFP control, GFP-tagged Trak1 WT or Trak1 hyrt in transfected HeLa cells. Anti-β-actin immunoblotting was used as a loading control. (B) Confocal microscopic analysis of HeLa cells expressing indicated GFP or GFP-tagged Trak1 proteins (green) with mitochondria labeled by MitoTracker Deep Red (MitoT; purple) and nuclei by DAPI (blue). Enlarged view of the boxed region is shown below the original image. Scale bars: 10 μm. (C) Mitochondrial morphology of cells was categorized based on the criteria of normal, hyperfused (containing elongated and/or enlarged mitochondria), elongated, or enlarged mitochondria. Mitochondria were considered elongated if they are greater than 3 μm in length and enlarged if they are greater than 1 μm in width. The percentage of transfected cells with indicated mitochondrial morphology was quantified and shown as mean ± SEM from three independent experiments. *, *P* < 0.05 versus the corresponding GFP control; #, *P* < 0.05 versus the corresponding Trak1 WT, one-way analysis of variance with a Tukey’s *post hoc* test. (D) 3D-SIM imaging analysis of HeLa cells expressing indicated GFP or GFP-tagged Trak1 proteins (green) with mitochondria labeled by DsRed2-Mito (DR-mito; red) or anti-TOM20 antibody (red). Enlarged view of the boxed region is shown below the original image. Scale bars: 2 μm. (E and F) The percentages of mitochondria with different ranges of mitochondrial length (E) or mitochondrial width (F) were quantified and shown as mean ± SEM of three independent experiments. In total, 851 (GFP), 571 (Trak1 WT), and 837 (Trak1 hyrt) mitochondria were analyzed. *, *P* < 0.05 versus the corresponding GFP control; #, *P* < 0.05 versus the corresponding Trak1 WT, one-way analysis of variance with a Tukey’s *post hoc* test

After finding that depletion of endogenous Trak1 causes fragmented mitochondrial morphology by reducing fusion activity, we performed experiments to determine whether expression of exogenous Trak1 WT or Trak1 hyrt can impact mitochondrial morphology. Immunofluorescence confocal microscopic analyses revealed that in HeLa cells containing endogenous Trak1, expression of GFP-tagged Trak1 WT, but not the GFP control, induced a mitochondrial hyperfusion phenotype with abnormally elongated and enlarged mitochondria (Fig. 4A–C). Quantification analysis showed that 91.0% ± 4.9% of Trak1 WT-expressing cells had hyperfused mitochondria (Fig. 4C). We found that the ability of exogenous Trak1 to induce mitochondrial hyperfusion was significantly reduced by hypertonia-linked mutation, as only 59.5% ± 8.6% of Trak1 hyrt-expressing cells had hyperfused mitochondria, with predominately elongated mitochondria rather than enlarged mitochondria (Fig. 4B and 4C).

Dual-color 3D-SIM super-resolution imaging analyses showed that Trak1 WT was targeted to the outer mitochondrial membrane of HeLa cells, as demonstrated by the colocalization of Trak1 WT with the OMM marker TOM20 that outlined DsRed2-Mito-labeled mitochondrial matrix (Fig. 4D). Abnormally elongated and enlarged mitochondria were observed in Trak1 WT-expressing cells but not in the GFP-expressing controls (Fig. 4D). Morphometric analysis indicated that both mitochondrial length (Fig. 4E) and mitochondrial width (Fig. 4F) were significantly increased by exogenous Trak1 WT expression. In agreement with our finding of hypertonia mutation-induced partial mislocalization of Trak1 from mitochondria to the cytosol (Fig. 3), super-resolution imaging analysis showed presence of Trak1 hyrt in both the OMM and cytosol (Fig. 4D). We found that Trak1 hyrt was capable of causing mitochondrial elongation, as shown by its ability to increase mitochondrial length to a similar extent as Trak1 WT (Fig. 4E). However, Trak1 hyrt was much less effective than Trak1 WT in causing mitochondrial enlargement, as demonstrated by the significantly reduced ability of Trak1 hyrt to increase mitochondrial width compared to Trak1 WT (Fig. 4F).

**Figure 5:**
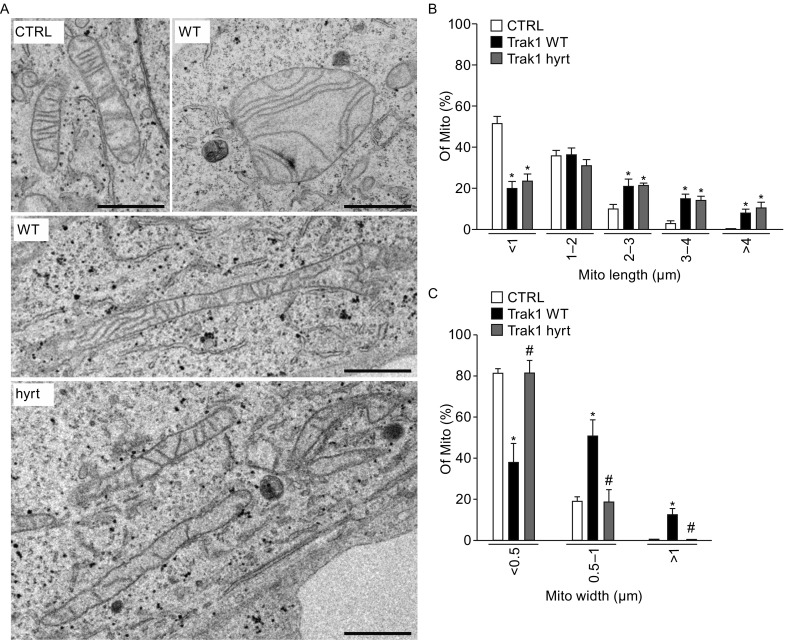
**Mitochondrial ultrastructural changes caused by exogenous Trak1 WT and Trak1 hyrt expression**. (A) Electron microscopic analysis of mitochondrial ultrastructure in GFP-tagged Trak1 WT- or Trak1 hyrt-transfected HeLa cells and mock-transfected controls (CTRL). Scale bars: 1 μm. (B and C) The percentages of mitochondria with different ranges of mitochondrial length (B) or mitochondrial width (C) were quantified and shown as mean ± SEM (*n* = 6). In total, 299 (CTRL), 157 (Trak1 WT), and 173 (Trak1 hyrt) mitochondria were analyzed. *, *P* < 0.05 versus the corresponding CTRL; #, *P* < 0.05 versus the corresponding Trak1 WT, one-way analysis of variance with a Tukey’s *post hoc* test

Next, we performed electron microscopy analyses to examine mitochondrial ultrastructural changes caused by exogenous Trak1 WT or Trak1 hyrt expression. We found that exogenous Trak1 WT expression resulted in formation of abnormally enlarged, oval-shaped mitochondria and abnormally long tubular mitochondria (Fig. 5A), which were absent in the control cells. These abnormal mitochondria had intact, outer and inner mitochondrial membranes, but their cristae structures were distorted and disorganized (Fig. 5A). Quantitative analysis indicated that exogenous Trak1 WT expression caused a significant increase in mitochondrial length (Fig. 5B) and mitochondrial width (Fig. 5C), consistent with our 3D-SIM results. In contrast, Trak1 hyrt-expressing cells contained mainly abnormally long tubular mitochondria with disorganized or disrupted cristae structures (Fig. 5A). We found that the mitochondrial length (Fig. 5B), but not the mitochondrial width (Fig. 5C), was significantly increased by Trak1 hyrt expression. Together, our confocal, 3D-SIM, and EM results indicate that Trak1 overexpression elongates and enlarges mitochondria, providing additional evidence supporting a role of Trak1 in facilitating mitochondrial fusion. Furthermore, our results indicate that hypertonia-associated mutation significantly impairs the ability of exogenous Trak1 to induce mitochondrial enlargement but not mitochondrial elongation.

### Trak1 interacts and colocalizes with mitofusins on the OMM and acts with mitofusins to promote mitochondrial fusion

**Figure 6:**
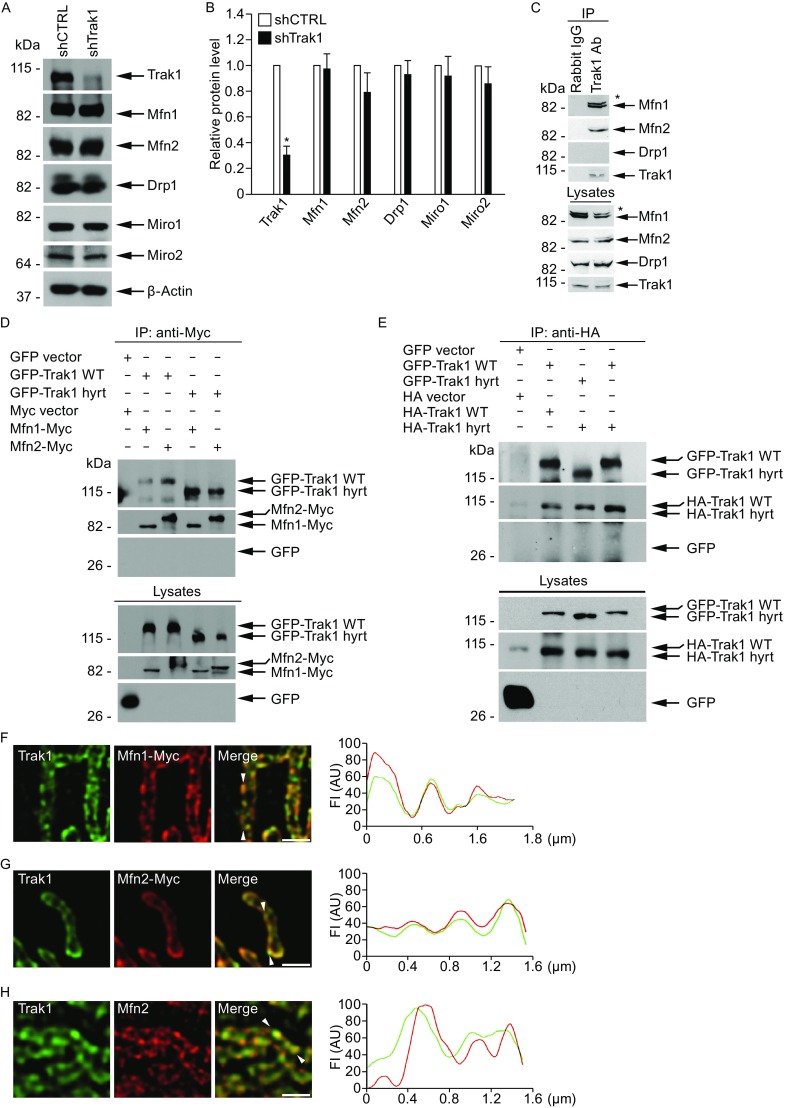
**Trak1 interacts and colocalizes with mitofusins on the OMM**. (A) Western blot analysis of lysates from shCTRL- or shTrak1-transfected HeLa cells using antibodies against Trak1, Mfn1, Mfn2, Drp1, Miro1, Miro2, and β-actin. (B) The relative protein level of Trak1 or indicated mitochondrial protein was normalized to the β-actin level in the corresponding cell lysate and expressed relative to the normalized protein level in the shCTRL cell lysate. Data represents mean ± SEM from three independent experiments. *, *P* < 0.05 versus the shCTRL control, unpaired two-tailed student’s *t* test. (C) Co-immunoprecipitation of endogenous Trak1 and mitofusins in cells. HeLa cell lysates were immunoprecipitated with anti-Trak1 antibody or rabbit IgG control followed by immunoblotting with antibodies against Trak1, Mfn1, Mfn2, and Drp1. The asterisk indicates a band that likely represents a posttranslational modified form of Mfn1. (D) Interaction of Trak1 WT or Trak1 hyrt with mitofusins in cells. Lysates from shTrak1 cells co-transfected with the indicated GFP-tagged and Myc-tagged constructs were subjected to immunoprecipitation with anti-Myc antibody followed by immunoblotting with anti-Myc and anti-GFP antibodies. (E) Homo-oligomerization of Trak1 in cells. Lysates from shTrak1 cells co-transfected with the indicated GFP-tagged and HA-tagged constructs were subjected to immunoprecipitation with anti-HA antibody followed by immunoblotting with anti-HA and anti-GFP antibodies. (F–H) 3D-SIM imaging analysis of transfected HeLa cells expressing indicated Myc-tagged Mfn1 (F) or Mfn2 (G) immunostained with anti-Myc (red) and anti-Trak1 (green) antibodies or untransfected HeLa cells immunostained with anti-Mfn2 (red) and anti-Trak1 (green) antibodies (H). Scale bars: 2 μm. Line scans show the fluorescence intensity profiles of each fluorescence signal along a line drawn through the OMM between the two arrowheads

Our finding of a role of Trak1 in mitochondrial fusion prompted us to examine the relationship between Trak1 and the mitochondrial fusion machinery components Mfn1 and Mfn2. Immunoblot analyses showed that depletion of endogenous Trak1 did not cause any significant change in the steady-state levels of Mfn1, Mfn2, or other mitochondrial proteins examined (Fig. 6A and 6B), suggesting that Trak1 does not have a role in regulation of protein levels of mitofusins. By using co-immunoprecipitation analyses, we found that endogenous Trak1 specifically interacted with Mfn1 and Mfn2 but not with Drp1 (Fig. 6C) and that the ability of Trak1 to interact with mitofusins was not affected by hypertonia-associated mutation (Fig. 6D). In agreement with a previous report (Koutsopoulos et al., 2010), our co-immunoprecipitation analyses showed that Trak1 has the ability to self-associate, indicating that Trak1 can undergo oligomerization in cells (Fig. 6E). We found that the self-association of Trak1 was not altered by hypertonia-associated mutation.

Previous studies have shown that mitofusins are localized in specific mitochondrial subdomains thought to represent potential sites of mitochondrial fusion (Karbowski et al., 2006; Neuspiel et al., 2005). To determine whether Trak1 colocalizes with mitofusins in these mitochondrial subdomains, we performed dual-color 3D-SIM super-resolution imaging analyses to compare the spatial distribution of endogenous Trak1 with that of Myc-tagged Mfn1 or Mfn2 on mitochondria. Consistent with previous reports (Karbowski et al., 2006; Neuspiel et al., 2005), Myc-tagged Mfn1 and Mfn2 were found to localize in discrete subdomains along the OMM (Fig. 6F and 6G). We observed extensive colocalization of endogenous Trak1 with Myc-tagged mitofusins in these subdomains of the OMM (Fig. 6F and 6G). Furthermore, although we were unable to find a reliable anti-Mfn1 antibody for immunostaining of endogenous Mfn1, we were able to perform double immunostaining 3D-SIM experiments with anti-Trak1 and anti-Mfn2 antibodies and found that endogenous Trak1 and Mfn2 proteins colocalize in the OMM subdomains (Fig. 6H). Together, these results provide evidence for the colocalization of Trak1 with mitofusins on the OMM at potential sites of mitochondrial fusion.

**Figure 7:**
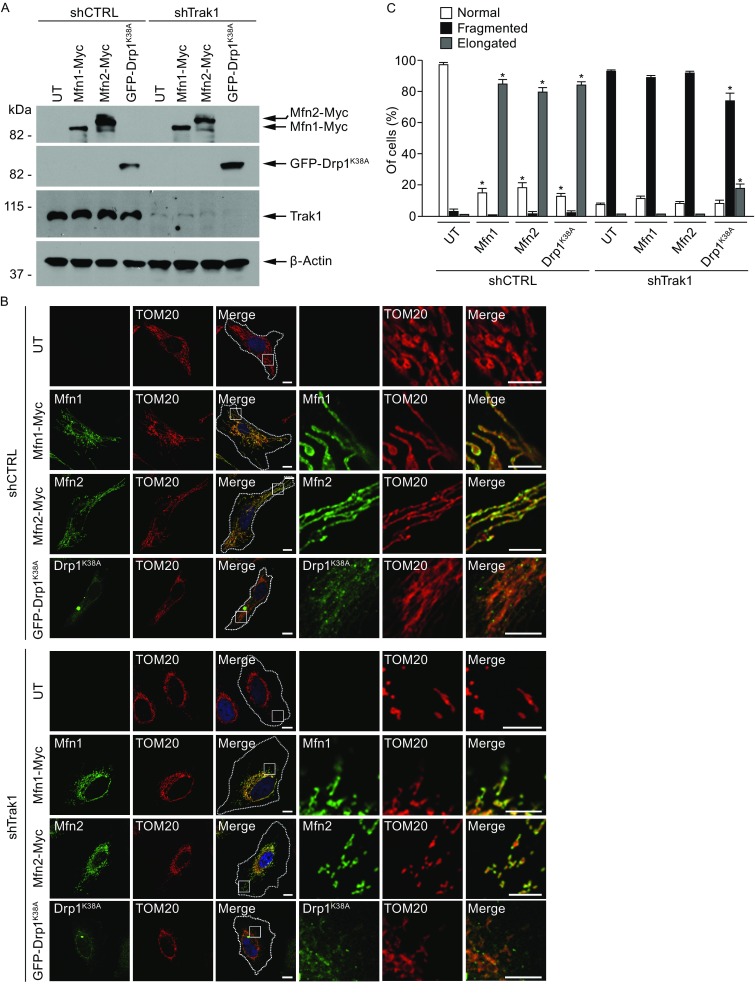
**Mitochondrial fragmentation due to loss of Trak1 cannot be reversed by mitofusin overexpression or Drp1** ^**K38A**^ **expression**. (A) Trak1-depleted HeLa cells (shTrak1) and control cells (shCTRL) were either untransfected (UT) or transfected with Myc-tagged Mfn1 or Mfn2, or GFP-tagged Drp1^K38A^ as indicated, and cell lysates were analyzed by immunoblotting with anti-Myc, anti-GFP, anti-Trak1, and anti-β-actin antibodies. (B) Confocal microscopic analysis of untransfected (UT) or transfected shCTRL and shTrak1 cells expressing Myc-tagged Mfn1 or Mfn2 (green), or GFP-tagged Drp1^K38A^ (green). Mitochondria were visualized by immunostaining with anti-TOM20 antibody (red). The boundary of cells is indicated by the dotted line and nuclei visualized by DAPI stain (blue) in merged images. Enlarged view of the boxed region is shown next to the original image. Scale bars: 10 μm. (C) The percentage of cells with indicated mitochondrial morphology was quantified and shown as mean ± SEM of three independent experiments. *, *P* < 0.05 versus the corresponding UT, one-way analysis of variance with a Tukey’s *post hoc* test

To investigate the functional relationship between Trak1 and the mitochondrial fusion and fission machinery, we performed experiments to test whether inhibiting mitochondrial fission with the dominant-negative Drp1^K38A^ mutant or promoting mitochondrial fusion by mitofusin overexpression can ameliorate the mitochondrial fragmentation phenotype of Trak1 depletion. We found that, although Drp1^K38A^ expression in control cells caused mitochondria to become elongated and interconnected due to unbalanced fusion (Smirnova et al., 2001; Smirnova et al., 1998), the ability of Drp1^K38A^ to cause mitochondrial elongation was greatly reduced by Trak1 depletion (Fig. 7), indicating endogenous Trak1 is required for mitochondrial fusion. Furthermore, we found that overexpression of Mfn1 or Mfn2 in control cells caused mitochondrial hyperfusion, resulting in mitochondria that are mostly elongated and occasionally enlarged (Fig. 7), consistent with previous reports (Legros et al., 2002; Rojo et al., 2002). However, in Trak1-depleted cells, overexpression of Mfn1 or Mfn2 was no longer able to promote mitochondrial fusion, and mitochondria remained fragmented (Fig. 7), indicating that endogenous Trak1 is required for mitofusin-mediated mitochondrial fusion. The inability of mitofusin overexpression to suppress mitochondrial fragmentation due to loss of Trak1 suggests that Trak1 acts with mitofusins in mitochondrial fusion.

### Trak1 promotes mitochondrial tethering in a mitofusin-independent manner

**Figure 8:**
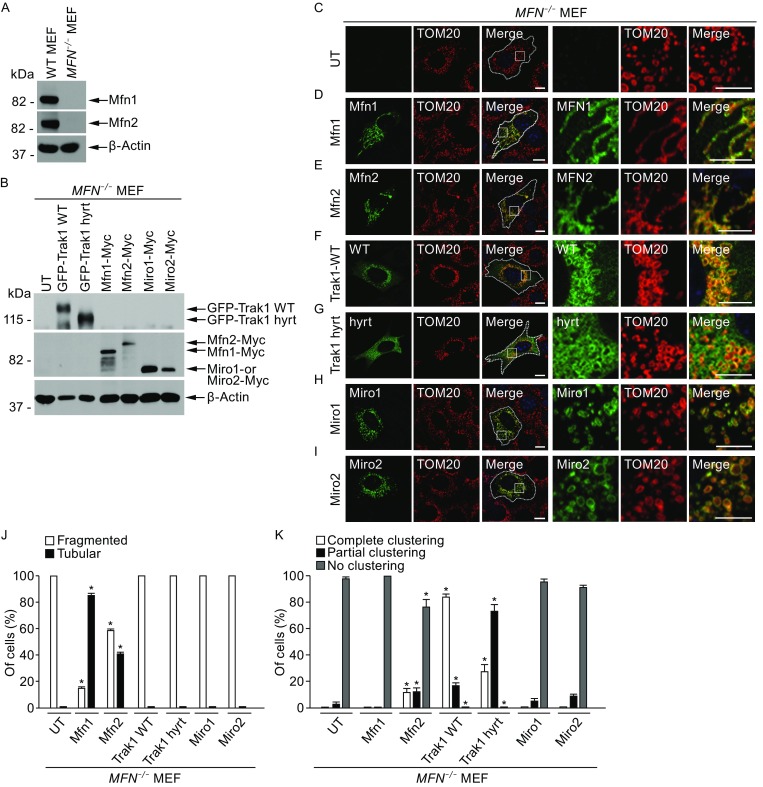
**Trak1 overexpression causes mitochondrial clustering in mitofusin-deficient cells**. (A) Western blot analysis of cell lysates from wild-type (WT) and *MFN* ^−/−^ MEFs with anti-Mfn1 and anti-Mfn2 antibodies shows the lack of both Mfn1 and Mfn2 proteins in *MFN* ^−/−^ MEFs. Anti-β-actin immunoblotting was used as a loading control. (B) Western blot analysis of cell lysates from untransfected (UT) or transfected *MFN* ^−/−^ MEFs with anti-Myc, anti-GFP, and anti-β-actin antibodies shows the expression of indicated exogenous proteins and β-actin in transfected *MFN* ^−/−^ MEFs. (C–I) Confocal microscopic analysis of untransfected (C) or transfected *MFN* ^−/−^ MEFs expressing Myc-tagged Mfn1 (D) or Mfn2 (E), GFP-tagged Trak1 WT (F) or Trak1 hyrt (G), Myc-tagged Miro1 (H) or Miro2 (I) after immunostaining with anti-TOM20 antibody (red) and anti-Myc antibody (green; D, E, H, and I). The boundary of cells is indicated by the dotted line and nuclei visualized by DAPI stain (blue) in merged images. Enlarged view of the boxed region is shown next to the original image. Scale bars: 10 μm in the original images and 5 μm in the enlarged images. (J) The percentage of transfected *MFN* ^−/−^ MEFs with fragmented or tubular mitochondrial morphology was quantified and compared to that of untransfected *MFN* ^−/−^ MEFs (UT). Data represent mean ± SEM of three independent experiments. *, *P* < 0.05 versus the corresponding UT, one-way analysis of variance with a Tukey’s *post hoc* test. (K) Mitochondrial clustering was analyzed by categorizing cells based on the criteria of complete clustering, partial clustering, or no clustering. The percentage of transfected *MFN* ^−/−^ MEFs with indicated mitochondrial clustering status was quantified and compared to that of UT cells. Data represent mean ± SEM of three independent experiments. *, *P* < 0.05 versus the corresponding UT, one-way analysis of variance with a Tukey’s *post hoc* test

To determine whether Trak1 has the ability to promote mitochondrial fusion in the absence of mitofusins, we used *Mfn1*/*Mfn2*-null mouse embryonic fibroblasts (*MFN* ^−/−^ MEFs) that lack expression of both Mfn1 and Mfn2 proteins (Fig. 8A). As reported previously (Chen et al., 2003, 2005), *MFN* ^−/−^ MEFs exhibited a mitochondrial fragmentation phenotype with small globular mitochondria scattered throughout the cytoplasm (Fig. 8C), a smilar phenotype is seen in HeLa cell depleted for endogenous Mfn proteins (Eura et al., 2003, 2006), supporting the conserved function of mitofusin proteins in HeLa cells and MEFs. The mitochondrial fragmentation phenotype of *MFN* ^−/−^ MEFs could be ameliorated by expression of exogenous Mfn1 (Fig. 8B, 8D, and 8J) or Mfn2 (Fig. 8B, 8E, and 8J), although Mfn1 was more effective than Mfn2 in restoring the tubular mitochondrial morphology (Fig. 8J). In contrast, despite the ability of Trak1 WT overexpression to cause mitochondrial hyperfusion in normal MEF cells (data not shown) similar to our results obtained in HeLa cells (Fig. 4), Trak1 WT overexpression was unable to ameliorate the mitochondrial fragmentation phenotype of *MFN* ^−/−^ MEF cells (Fig. 8F and 8J), indicating that Trak1-driven mitochondrial fusion requires mitofusins. Interestingly, Trak1 WT overexpression resulted in extensive clustering of mitochondria in *MFN* ^−/−^ MEFs (Fig. 8F and 8K). We found that the ability of exogenous Trak1 to promote mitochondrial clustering was significantly impaired by Trak1 hyrt mutation (Fig. 8G and 8K). Furthermore, overexpression of mitochondrial Rho GTPase Miro1 (Fig. 8H), or Miro2 (Fig. 8I), a family of Trak1-binding proteins which facilitates transport of mitochondria along microtubules (Macaskill et al., 2009; Saotome et al., 2008), was incapable of causing mitochondrial clustering or suppressing mitochondrial fragmentation in *MFN* ^−/−^ MEFs (Fig. 8J and 8K). These data, together with previous reports of enhanced mitochondrial motility by Miro1 or Miro2 overexpression (Macaskill et al., 2009; Saotome et al., 2008), suggest that the ability to induce mitochondrial clustering is a unique function of Trak1 that can be uncoupled from the effect on mitochondria transport.

**Figure 9:**
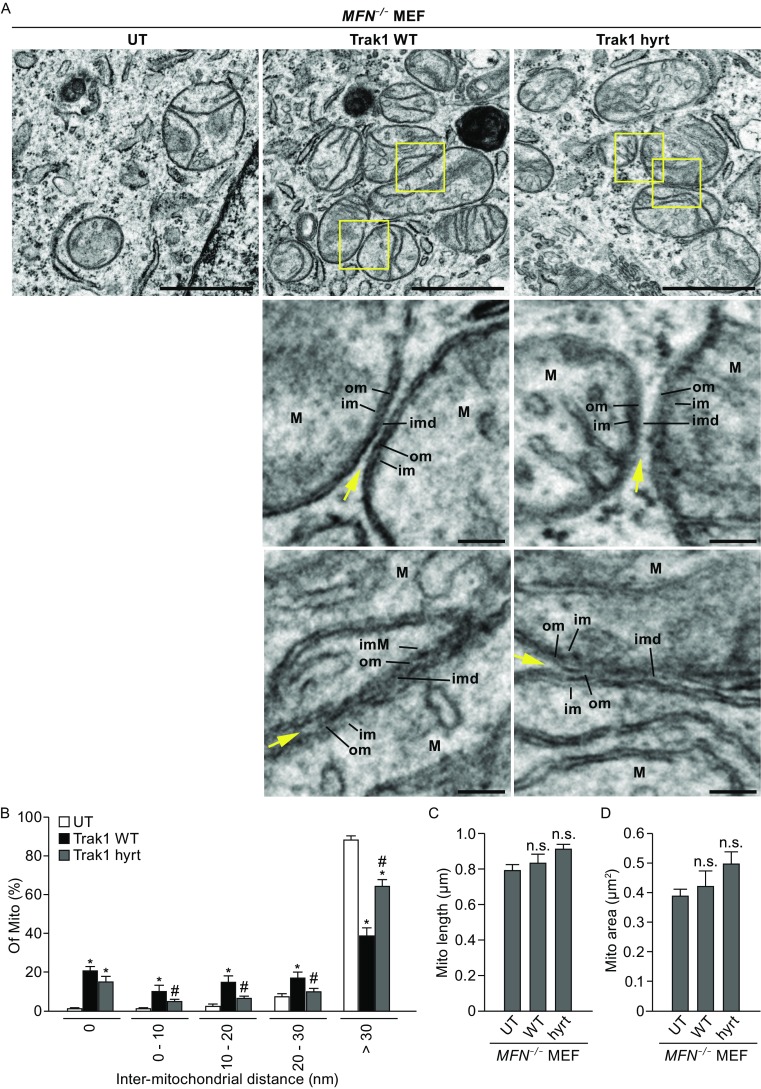
**Trak1 promotes mitochondrial tethering in the absence of mitofusins**. (A) Electron microscopic analysis of mitochondrial structures in untransfected (UT), GFP-tagged Trak1 WT- or Trak1 hyrt-transfected *MFN* ^−/−^ MEFs. Enlarged views of the boxed regions are shown below the original image. Arrow indicates the gap between the opposing outer membranes of adjacent mitochondria. Scale bars: 1 μm in the original images and 100 nm in the enlarged images. M, mitochondrion; om, outer mitochondrial membrane; im, inner mitochondrial membrane; imd, inter-mitochondrial distance. (B–D) The percentages of mitochondria with different ranges of inter-mitochondrial distance (B), mitochondrial area (C), and mitochondrial length (D) were quantified and shown as mean ± SEM (*n* = 11). In total, 320 (UT), 258 (Trak1 WT), and 269 (Trak1 hyrt) mitochondria pairs were analyzed. *, *P* < 0.05 versus the corresponding UT; #, *P* < 0.05 versus the corresponding Trak1 WT; n.s., not significant versus the corresponding UT or Trak1 WT; one-way analysis of variance with a Tukey’s *post hoc* test

Membrane fusion is a multi-step process that starts with a tethering step to juxtapose opposing membranes in close distance (~30 nm), followed by a docking step to bring two membranes within a bilayer’s distance of one another (<5–10 nm) before the final fusion of the bilayers occurs (Pfeffer, 1999; Brocker et al., 2010). Previous studies have shown that, when mitochondrial fusion was inhibited by removal of Mfn1 GTPase domain, mitochondria were trapped in a tethered state mediated by the HR2 coiled coil region of Mfn1, which appeared as mitochondrial clustering in confocal microscopic images (Koshiba et al., 2004). Interestingly, the mitochondrial clustering induced by Trak1 WT overexpression in *MFN* ^−/−^ MEFs (Fig. 8F) looked similar to that induced by expression of truncated Mfn1 containing the HR2 region (Koshiba et al., 2004), suggesting that Trak1 WT may promote mitochondrial tethering in *MFN* ^−/−^ MEFs. To examine this possibility, we performed EM analyses to further characterize the effects of exogenous Trak1 WT expression on mitochondria in *MFN* ^−/−^ MEFs. We found that, in contrast to mitochondria in *MFN* ^−/−^ MEFs which were well separated spatially (Fig. 9A), mitochondria in Trak1 WT-expressing *MFN* ^−/−^ MEFs were clustered with opposing mitochondrial outer membranes in close juxtaposition (Fig. 9A). At higher magnification, the outer membranes of some neighboring mitochondria seemed to be connected in several regions through electron-dense materials, while in other cases, the outer membranes of adjacent mitochondria appeared to be in direct contact with one another at restricted domains (Fig. 9A). Quantification of the distances between opposing outer membranes of adjacent mitochondria showed that a majority of mitochondria in Trak1 WT-expressing *MFN* ^−/−^ MEFs were in a tethered or docked state with an inter-mitochondrial distance of less than 30 nm (Fig. 9B). In contrast, mitochondria in *MFN* ^−/−^ MEFs had much larger inter-mitochondrial distances, and few mitochondria were found in a tethered or docked state (Fig. 9B). Trak1 WT-induced mitochondrial apposition in *MFN* ^−/−^ MEFs exhibited an average distance of 12.3 ± 2.9 nm between the outer membranes, which is similar to the reported distance of 15.9 ± 3.0 nm for Mfn1-mediated mitochondrial tethering (Koshiba et al., 2004). Taken together, our results indicate that Trak1 WT is capable of facilitating mitochondrial tethering in a mitofusin-independent manner. Parallel analysis of the effects of Trak1 hyrt expression in *MFN* ^−/−^ MEFs showed that the ability of Trak1 to promote mitochondrial tethering was significantly impaired by Trak1 hyrt mutation (Fig. 9A and 9B). Consistent with confocal microscopy data (Fig. 8F, 8G, and 8J), EM analysis revealed no significant effects of Trak1 WT or Trak1 hyrt expression on mitochondrial length (Fig. 9C) or mitochondrial size (Fig. 9D) in *MFN* ^−/−^ MEFs, indicating Trak1 is unable to promote mitochondrial fusion in the absence of mitofusins.

### Trak1 is essential for stress-induced mitochondrial hyperfusion and pro-survival response

**Figure 10:**
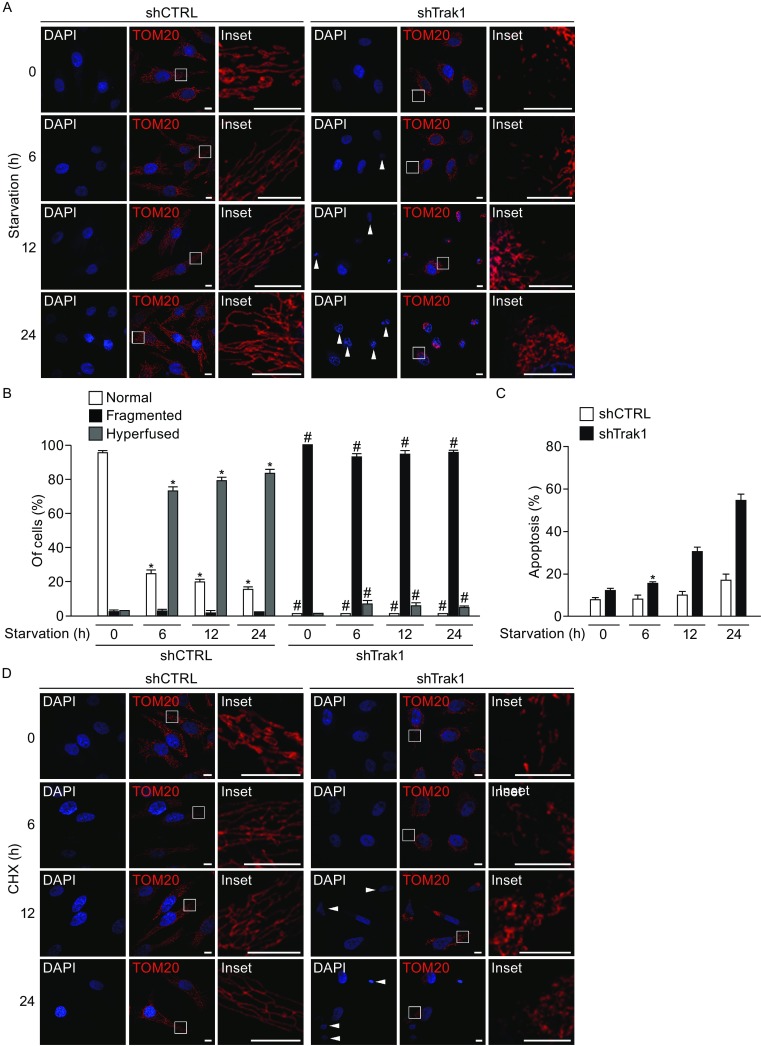

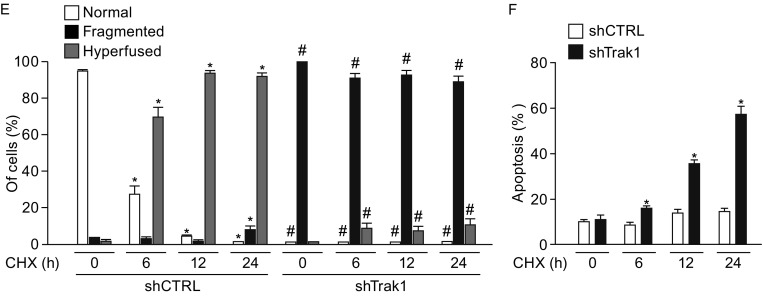
**Stress-induced mitochondrial hyperfusion and pro-survival response require Trak1**. (A and D) Confocal microscopic analysis of stable shCTRL- and shTrak1-transfected HeLa cells after starvation with HBSS (A) or exposure to protein synthesis inhibitor CHX (D) for the indicated lengths of time. Mitochondria were visualized by immunostaining with anti-TOM20 antibody (red), and nuclear integrity was assessed by DAPI staining (blue). Inset shows an enlarged view of the boxed region, and arrowheads indicate apoptotic nuclei. Scale bars: 10 μm. (B and E) The percentage of cells with indicated mitochondrial morphology was quantified at the indicated time points after stress exposure and shown as mean ± SEM of three independent experiments. *, *P* < 0.05 versus the shCTRL-transfected control at 0 h; #, *P* < 0.05 versus the corresponding shCTRL control at the same time point, one-way analysis of variance with a Tukey’s *post hoc* test. (C and F) Apoptosis was quantified as the percentage of cells with apoptotic nuclear morphology and shown as mean ± SEM of three independent experiments. *, *P* < 0.05 versus the shCTRL-transfected control at 0 h, one-way analysis of variance with a Tukey’s *post hoc* test

Recent evidence indicates that mitochondria undergo hyperfusion in response to cellular stress such as nutrient starvation or protein synthesis inhibition, and this stress-induced mitochondrial hyperfusion has a cytoprotective role in cell survival under stress conditions (Gomes et al., 2011; Rambold et al., 2011; Tondera et al., 2009). To determine whether Trak1 functions in mitochondrial hyperfusion during cellular stress, we first examined the effects of Trak1 depletion on starvation-induced mitochondrial hyperfusion. We found that, whereas mitochondria in control cells elongated or hyperfused after starvation with HBSS, mitochondria in Trak1-depleted cells were incapable of undergoing starvation-induced mitochondrial elongation, and they remained fragmented (Fig. 10A and 10B). Similar analyses revealed that Trak1 depletion also abolished the ability of mitochondria to undergo hyperfusion in response to protein synthesis inhibition by CHX (Fig. 10D and 10E). Together, these results indicate that endogenous Trak1 is required for stress-induced mitochondrial hyperfusion. Assessment of the extent of cell death under stress conditions showed that the inability of Trak1-depleted cells to undergo stress-induced mitochondrial hyperfusion was accompanied by increased sensitivity to starvation- or CHX-induced cell death (Fig. 10), indicating an essential role of Trak1 in cellular defense against stress by promoting mitochondrial fusion.

## DISCUSSION

This study reveals a new role for Trak1 as a regulator of mitochondrial fusion. We found that endogenous Trak1 is required for normal morphogenesis of mitochondria by controlling mitochondrial fusion. Depletion of Trak1 in cells decreases mitochondrial fusion rate, resulting in a mitochondrial fragmentation phenotype with shorter and smaller mitochondria. Conversely, increasing Trak1 protein level in cells causes a mitochondrial hyperfusion phenotype with elongated and enlarged mitochondria. Our results indicate that Trak1 has the ability to actively promote mitochondrial fusion.

Diverse membrane fusion processes occur in cells through a common set of steps: membrane tethering, docking, and fusion (Brocker et al., 2010; Li and Chin, 2003). Tethering factors for many intracellular membrane fusion processes have been identified and shown to not only act as physical bridges to connect two opposing membranes but also interact with multiple components of the fusion machinery to promote docking and SNARE-mediated membrane fusion (Brocker et al., 2010; Yu and Hughson, 2010). In contrast, little is known about the tethering factors and molecular mechanism for mitochondrial fusion. Current models propose that mitochondrial OMM-localized GTPases Mfn1 and Mfn2 mediate mitochondrial tethering through formation of homo-oligomeric or hetero-oligomeric complexes between adjacent mitochondria and use GTP hydrolysis-induced conformational changes to drive mitochondrial OMM fusion (Chan, 2012; Pernas and Scorrano, 2016). Our results indicate that Trak1 interacts and colocalizes with Mfn1 and Mfn2 on the OMM and that Trak1 is required for mitofusin-mediated mitochondrial fusion. Interestingly, our analyses reveal that Trak1 is capable of undergoing homo-oligomerization in cells and has the ability to mediate mitochondrial tethering in a mitofusin-independent manner. However, Trak1 is unable to promote mitochondrial fusion in the absence of mitofusins. Together, our findings support a function of Trak1 as a tethering factor that acts with mitofusins to promote mitochondrial tethering and Mfn-mediated OMM fusion.

Mitochondrial fusion plays a critical role in the maintenance of mitochondrial and cellular homeostasis (Chan, 2012; Pernas and Scorrano, 2016). Recent studies have shown that, in response to a variety of cellular stresses, mitochondria elongate and hyperfuse to sustain mitochondrial function and prevent apoptotic cell death (Gomes et al., 2011; Rambold et al., 2011; Tondera et al., 2009). Although the molecular mechanism underlying stress-induced mitochondrial hyperfusion is poorly understood, recent evidence indicates that this mitochondrial hyperfusion process requires Mfn1, OPA1, and OPA1-regulating protein SLP-2, but not Mfn2 or Mfn2-regulating proteins Bax and Bak (Gomes et al., 2011; Rambold et al., 2011; Tondera et al., 2009). Our finding that depletion of Trak1 not only abolishes the ability of mitochondria to hyperfuse but also reduces cell survival under stress conditions reveals an essential role of Trak1 in mediating stress-induced mitochondrial hyperfusion and pro-survival response.

The importance of mitochondrial fusion to human health is underscored by the findings that mutations in the mitochondrial fusion machinery components Mfn2 and OPA1 cause Charcot-Marie-Tooth disease type 2A and autosomal dominant optic atrophy, respectively (Zuchner et al., 2004; Alexander et al., 2000; Delettre et al., 2000). Genetic analyses have identified a homozygous Trak1 mutation resulting in a C-terminal truncated form of Trak1 as the cause of recessively inherited hypertonia (Gilbert et al., 2006), but the pathogenic mechanism of Trak1 mutation remains unknown. We found that hypertonia-associated mutation impairs Trak1 mitochondrial localization and its ability to facilitate mitochondrial tethering and fusion. Our results indicate a link between dysregulated mitochondrial fusion and hypertonia pathogenesis.

In summary, this study uncovers a function of Trak1 as a novel regulator of mitochondrial fusion, acting upstream of mitofusins to promote mitochondrial tethering and fusion. Our work reveals that Trak1 participates in stress-induced mitochondrial hyperfusion and promotes cell survival under stress conditions. Furthermore, our finding of impairment of Trak1-mediated mitochondrial fusion by hypertonia-associated mutation provides new insights into the pathogenic mechanism of hypertonia. Based on our results, we suggest that enhancement of Trak1-mediated mitochondrial fusion could represent a novel therapeutic strategy to combat mitochondrial fragmentation in a number of neurodegenerative diseases.

## MATERIALS AND METHODS

### Expression constructs

The expression constructs encoding N-terminal GFP-tagged human Trak1 WT (residues 1–953) and Trak1 hyrt (residues 1–824) were generated as previously described (Webber et al., 2008). The rescue expression constructs encoding shRNA-resistant GFP-tagged Trak1 WT and Trak1 hyrt were generated by site-directed mutagenesis to make two or three silent third-codon substitutions within the shRNA-targeted region of the Trak1 transcript without altering the Trak1 amino acid sequence. The full-length Miro1 and Miro2 expression constructs were provided by Dr. Pontus Aspenstrom (Ludwig Institute for Cancer Research, Uppsala University, Sweden), and full-length Mfn1 and Mfn2 constructs by Dr. David Chan (California Institute of Technology). The DsRed2-Mito plasmid for expressing mitochondrial matrix-targeted red fluorescent protein DsRed2 was obtained from (Clontech), and the mito-Dendra2 construct was a gift from Dr. Michael T. Ryan (La Trobe University, Australia). The shRNA constructs targeting human Trak1 (NM_014965.2-876s1c1 and NM_014965.2-1392s1c1) and a non-targeting shRNA control construct (SHC001) were from Sigma-Aldrich.

### Antibodies

Rabbit polyclonal anti-Trak1 antibody was generated against the synthetic peptide corresponding to residues 935–953 of human Trak1 and was affinity-purified as previously described (Webber et al., 2008). Other primary antibodies used in this study include: anti-TOM20 (Santa Cruz); anti-Mfn1 (Abcam); anti-Mfn2 (ProteinTech Group, Inc.); anti-Miro1 (clone 4H4, Abnova); anti-Miro2 (ProteinTech Group, Inc); anti-Drp1 (Abcam); anti-GFP (B2, Santa Cruz); anti-Myc (9E10); anti-HSP60 (Stressgen); anti-GAPDH (Cell Signaling); and anti-β-actin (clone C4, Millipore). Horseradish-peroxidase-conjugated and FITC- or TRITC-conjugated secondary antibodies were from Jackson ImmunoResearch Laboratories.

### Cell culture and transfection

HeLa cells (ATCC CCL-2^TM^), wild-type mouse embryonic fibroblasts (WT MEFs; ATCC CRL-2991^TM^), and *Mfn1*/*Mfn2*-null MEFs (ATCC CRL-2994^TM^) were obtained from American Type Culture Collection (ATCC). Cells were grown in Dulbecco’s modified Eagle medium (GIBCO) with 10% (*v*/*v*) fetal bovine serum (Atlanta Biologicals) and 1% (*v*/*v*) penicillin-streptomycin (Fisher) in a humidified incubator at 37°C with 5% CO_2_. HeLa cells and MEF cells were transfected with the indicated plasmids using Lipofectamine 2000 reagent (Invitrogen) and Fugene HD (Promega), respectively, according to the manufacturer’s instructions. For generation of stable shTrak1 and shCTRL cell lines, Trak1 shRNA- or non-targeting control shRNA-transfected HeLa cells were selected with 2.5 μg/mL puromycin (Research Products International), and single puromycin-resistant colonies were isolated for culture.

### Immunoblotting analysis

Cells were homogenized in 1% SDS, and protein extracts were analyzed by SDS-PAGE and subsequent immunoblotting with the indicated primary antibodies and horseradish-peroxidase-conjugated second antibodies followed by visualization using enhanced chemiluminescence as described previously (Webber et al., 2008). For quantification of relative levels of mitochondrial protein in stable shCTRL and shTrak1 cells, equal amounts of protein from each cell lysate were subjected to immunoblotting, and the band intensity of each protein on immunoblot images was quantified by using the Image J software (National Institutes of Health) and normalized to the corresponding band intensity of β-actin.

### Co-immunoprecipitation analysis

Cell lysates were prepared with lysis buffer (50 mmol/L Tris-HCl, pH 7.6, 100 mmol/L NaCl, 1% IGEPAL CA-630, 0.1% Triton-X-100, and a cocktail of protease inhibitors) as described (Chin et al., 2001), and the clarified supernatants were incubated with the indicated antibody, either anti-Trak1 (rabbit polyclonal antibody), rabbit serum IgG, or anti-Myc (9E10) for 4 h at 4°C. Recovery of immunocomplexes was achieved using protein G-Sepharose beads (EMD Millipore). After multiples washes, the immunoprecipitated protein complexes were analyzed by SDS-PAGE and immunoblotting.

### Immunofluorescence confocal microscopy

For immunostaining, cells were grown on poly-L-lysine-coated coverslips, fixed in 4% paraformaldehyde for 20 min, and permeabilized with a solution containing 0.1% saponin (Sigma-Aldrich) and 4% horse serum in PBS. Cells were stained with the indicated primary and secondary antibodies and processed for immunofluorescence confocal microscopy as we described previously (Lee et al., 2012). For mitochondrial labeling, MitoTracker Deep Red FM (Life Technologies) was added to living cells at a final concentration of 25 nmol/L and incubated for 15 min and then washed with pre-warmed media for 30 min at 37°C before fixation. Nuclei were visualized with 4’,6-diamidino-2-phenylindole (DAPI) according to the instructions of the manufacturer (Life Technologies). Image acquisition was conducted using a Nikon Eclipse Ti confocal laser-scanning microscope as previously described (Lee et al., 2012). Images were exported in TIFF format with the Nikon EZ-C1 viewer software and processed using Adobe Photoshop CS5 software (Adobe Systems, Inc) to produce the figures.

### Three-dimensional structured illumination microscopy (3D-SIM)

Cells were fixed and processed as we described previously (Fallaize et al., 2015). Images were captured using a CFI Apochromat TIRF 100×/1.49 NA oil immersion objective lens on an N-SIM microscope (Nikon Instruments Inc., Melville, NY) as described (Fallaize et al., 2015; Lee et al., 2012). In each z plane, 15 images were acquired with a rotating illumination pattern (5 phases and 3 angles) in two color channels (488 nm and 561 nm) independently, using the following parameters: structured illumination contrast = 2.0; apodization filter = 1.0; width of 3D-SIM filter = 0.20. Image acquisition and reconstruction were performed using NIS-Elements software (Nikon Instruments, Melville, NY).

### Electron microscopy

For ultrastructural analysis, cells were fixed with 2.5% glutaraldehyde in 0.1 mol/L cacodylate buffer (pH 7.4) followed by post-fixation with 1% osmium and 1.5% potassium ferrocyanide in the same buffer. Cells were then dehydrated in ethanol and embedded in Eponate 12 resin. Ultrathin (70 nm) sections were cut with an ultramicrotome and stained with 5% uranyl acetate and 2% lead citrate. Images were acquired using a Hitachi H-7500 transmission electron microscope equipped with a SIA L12C 16 megapixel CCD camera.

### Analysis of mitochondrial morphology and clustering

Mitochondria were labeled with MitoTracker Deep Red FM or anti-TOM20 antibody and examined using the Nikon Eclipse Ti confocal microscope. Quantitative analysis of mitochondrial morphology was performed, as described previously (Mishra et al., 2014; Wang et al., 2009), by categorizing cells according to the following criteria: Normal, a mixed population of interconnected and non-connected, long and short tubular mitochondria; Fragmented, the majority of mitochondria were non-connected, small and spherical; Elongated, the majority of mitochondria were long tubular with the length of greater than 3 μm; Enlarged, abnormally large mitochondria with the width of greater than 1 μm. For each experiment, 25–50 cells per group per condition were randomly selected to quantify the percentage of cells with indicated mitochondrial morphology, and the experiment was independently performed three times. For analysis of mitochondrial clustering, cells were scored based on the following criteria: Complete clustering, nearly all mitochondria in the cell were closely juxtaposed; Partial clustering, some of mitochondria in the cell were juxtaposed; No clustering, mitochondria are scattered and not juxtaposed. The percentage of cells with indicated mitochondrial clustering status was quantified from 35–45 randomly selected cells per group for each experiment, and the experiment was repeated three times.

### Quantification of mitochondria size and mitochondrial tethering

To quantify mitochondria size in 3D-SIM and EM images, the length, width, and area of individual mitochondria were measured using the Image J software. For quantification of mitochondria size by 3D-SIM, morphometric analyses of mitochondrial length, width, and area were performed from at least three randomly selected cells (120–270 mitochondria) per group for each experiment, and the experiments were repeated three times. For quantification of mitochondria size by EM, morphometric analyses of mitochondrial length, width, and area were performed from four randomly selected fields per cell with at least 5 cells found in different EM grid sections (80–290 mitochondria) analyzed per group. To evaluate mitochondrial tethering, the distances between opposing mitochondrial outer membranes of adjacent mitochondria were measured using the Image J software on EM images at 20,000× magnification from at least eleven randomly selected cells found in different EM grid sections (250–320 mitochondria pairs) per group.

### Quantification of Trak1 colocalization with mitochondrial proteins

For analysis of colocalization using confocal microscopy, all images were acquired under identical settings using the Nikon Eclipse Ti confocal microscope. Quantification of the colocalization of endogenous Trak1, GFP-tagged Trak1 WT or Trak1 hyrt with the mitochondrial marker TOM20 was performed on unprocessed images as described (Lee et al., 2012). Single cells were selected by manually tracing cell outlines, the background was subtracted in each channel, and the fraction of Trak1 (or GFP) overlapping with TOM20 was determined by Mander’s colocalization coefficient using the NIS-Elements software (Nikon Instruments, Melville, NY). The fraction of colocalization was averaged from 25–40 randomly selected cells per group for each experiment, and three independent experiments were performed. Graphs were made with SigmaPlot 11.0 software, and Adobe Photoshop CS5 was used to produce figures.

### Live-cell imaging analyses of mitochondrial fusion and fission

Cells cultured in glass-bottom MatTek dishes were transfected with mitoDendra2. At 24 h post-transfection, cells were placed in an environmentally controlled chamber (37°C, 5% CO_2_ and humidity). Live-cell imaging was performed using a Nikon Eclipse Ti confocal microscope. A small subset of mitoDendra2-labeled mitochondria was irreversibly converted from green fluorescence (excitation at 488 nm and emission at 515 nm) to red fluorescence (excitation at 561 nm and emission at 590 nm) by photoactivation with the 408 nm laser at 2% intensity for 10–15 iterations. Time-lapse images were captured every 30 s for at least 20 min with the 488 nm laser at 0.1% intensity and the 561 nm laser at 0.5% intensity to prevent photobleaching. The extent of mitochondrial fusion at the indicated time points after photoactivation was quantified by measuring the colocalization of mitochondrial green and red fluorescence using Mander’s colocalization coefficient with the Image J software as described (Magrane et al., 2012; Lee et al., 2012) to determine the percentage of the area of green mitochondria overlapping with red mitochondria. The data were subjected to linear regression analysis to obtain the slope for calculation of the relative fusion rate. Mitochondrial fusion rate (fusion/mito/min) and fission rate (fusion/mito/min) were determined by counting the number of fusion events and number of fission events, respectively, that involve red mitochondria and occurred within 4 min after photoactivation and the obtained numbers were divided by the total number of red mitochondria at t = 0 min and by the duration of time (4 min).

### Subcellular fractionation

Cells were subjected to subcellular fractionation to obtain mitochondria and cytosol fractions described previously (Lee et al., 2011; Lazarou et al., 2012). Briefly, cells were homogenized in homogenization buffer (250 mmol/L sucrose, 20 mmol/L HEPES, pH 7.4, 10 mmol/L KCl, 1.5 mmol/L MgCl_2_, 0.1 mmol/L EDTA, 1 mmol/L EGTA, and protease inhibitors) with a Dounce homogenizer, and cell homogenates were centrifuged at 1000 ×*g* to remove nuclei and unbroken cells. The post-nuclear supernatant was subsequently centrifuged at 10,000 ×*g* for 15 min to pellet mitochondria. Mitochondria and cytosol fractions were analyzed along with the post-nuclear supernatant by SDS-PAGE and immunoblotting. The level of Trak1 in each fraction relative to the total level in the post-nuclear supernatant was quantified by measuring the intensity of the Trak1 band on immunoblot images using the Image J software as described previously (Lee et al., 2011; Giles et al., 2009).

### Analysis of stress-induced mitochondrial hyperfusion

Mitochondrial hyperfusion was induced by nutrient starvation with Hank’s balanced salt solution (HBSS; Life Technologies) or treating cells with 10 μmol/L cycloheximide (CHX; Sigma-Aldrich) for 0, 6, 12, and 24 h as previously described (Rambold et al., 2011; Tondera et al., 2009). At the indicated time points, cells were immunostained with anti-TOM20 antibody and stained with DAPI followed by analysis using fluorescence confocal microscopy. Mitochondrial morphology was scored as follows: Normal, a mixed population of interconnected and non-connected, long and short tubular mitochondria; Hyperfused: mitochondria were elongated and highly interconnected, with few non-connected mitochondria; Fragmented, the majority of mitochondria were non-connected, small and spherical. The percentage of cells with indicated mitochondrial morphology was quantified from 100–200 randomly selected cells per group for each experiment, and three independent experiments were performed.

### Apoptosis assay

The extent of apoptotic cell death was determined by morphological analysis of DAPI-stained nuclei to assess nuclear integrity as described previously (Chen et al., 2010). Nuclei with nuclear shrinkage, fragmentation, and chromatin condensation were scored as apoptotic nuclei. For each experiment, 100–200 cells per group per condition were randomly selected to quantify the percentage of cells with apoptotic nuclei, and the experiment was conducted a total of three times.

### Statistical analyses

Data were analyzed by unpaired two-tailed Student’s t test or a one- or two-way analysis of variance (ANOVA) followed with a Tukey’s *post hoc* test using the SigmaPlot software (Systat Software, Inc.). Results are expressed as mean ± SEM. A P value of < 0.05 was considered statistically significant.
